# RNA editing of human microRNAs

**DOI:** 10.1186/gb-2006-7-4-r27

**Published:** 2006-04-04

**Authors:** Matthew J Blow, Russell J Grocock, Stijn van Dongen, Anton J Enright, Ed Dicks, P Andrew Futreal, Richard Wooster, Michael R Stratton

**Affiliations:** 1 Cancer Genome Project, Wellcome Trust Sanger Institute, Wellcome Trust Genome Campus, Hinxton, Cambridge, CB10 1SA, UK; 2 Computational and Functional Genomics, Wellcome Trust Sanger Institute, Wellcome Trust Genome Campus, Hinxton, Cambridge, CB10 1SA, UK; 3 Section of Cancer Genetics, Institute of Cancer Research, Sutton, Surrey, SM2 5NG, UK

## Abstract

**Background:**

MicroRNAs (miRNAs) are short RNAs of around 22 nucleotides that regulate gene expression. The primary transcripts of miRNAs contain double-stranded RNA and are therefore potential substrates for adenosine to inosine (A-to-I) RNA editing.

**Results:**

We have conducted a survey of RNA editing of miRNAs from ten human tissues by sequence comparison of PCR products derived from matched genomic DNA and total cDNA from the same individual. Six out of 99 (6%) miRNA transcripts from which data were obtained were subject to A-to-I editing in at least one tissue. Four out of seven edited adenosines were in the mature miRNA and were predicted to change the target sites in 3' untranslated regions. For a further six miRNAs, we identified A-to-I editing of transcripts derived from the opposite strand of the genome to the annotated miRNA. These miRNAs may have been annotated to the wrong genomic strand.

**Conclusion:**

Our results indicate that RNA editing increases the diversity of miRNAs and their targets, and hence may modulate miRNA function.

## Abstract 2

A survey of RNA editing of miRNAs from ten human tissues indicates that RNA editing increases the diversity of miRNAs and their targets.

## Background

MicroRNAs (miRNAs) are short (around 20-22 nucleotides) RNAs that post-transcriptionally regulate gene expression by base-pairing with complementary sequences in the 3' untranslated regions (UTRs) of protein-coding transcripts and directing translational repression or transcript degradation [1-5]. There are currently 326 human miRNAs listed in the miRNA registry version 7.1 [6], but the total number of miRNAs encoded in the human genome may be nearer 1,000 [7,8]. The function of most miRNAs is unknown, but many are clearly involved in regulating differentiation [9] and development [10]. It is estimated that up to 30% of human genes may be miRNA targets [11,12].

miRNAs are transcribed by RNA polymerase II into long primary miRNA (pri-miRNA) transcripts which are capped and polyadenylated [13,14]. Genomic analyses indicate that many miRNAs overlap known protein coding genes or non-coding RNAs [15], and that many are in evolutionarily conserved clusters with other miRNAs [16]. Furthermore, intronic miRNAs share expression patterns with adjacent miRNAs and the host gene mRNA indicating that they are coordinately coexpressed [17].

Pri-miRNAs contain a short double-stranded RNA (dsRNA) stem-loop formed between the miRNA sequence and its adjacent complementary sequence. In the nucleus, the ribonuclease III-like enzyme Drosha cleaves at the base of this stem-loop to liberate a miRNA precursor (pre-miRNA) as a 60-70-nucleotide RNA hairpin [18]. The pre-miRNA hairpin is exported to the cytoplasm by exportin-5 [19-21] where it is further processed into a short dsRNA molecule by a second ribonuclease III-like enzyme, Dicer [22-24]. A single strand of this short dsRNA, the mature miRNA, is incorporated into a ribonucleoprotein complex. This complex directs transcript cleavage or translational repression depending on the degree of complementarity between the miRNA and its target site.

RNA editing is the site-specific modification of an RNA sequence to yield a product differing from that encoded by the DNA template. Most RNA editing in human cells is adenosine to inosine (A-to-I) RNA editing which involves the conversion of A-to-I in dsRNA [25,26]. A-to-I RNA editing is catalyzed by the adenosine deaminases acting on RNA (ADARs). The majority of A-to-I RNA-editing sites are in dsRNA structures formed between inverted repeat sequences in intronic or intergenic RNAs [25,27-30]. Therefore, the double-stranded precursors of miRNAs may be substrates for A-to-I editing. Indeed, it has recently been shown that the pri-miRNA transcript of human miRNA miR-22 is subject to A-to-I RNA editing in a number of human and mouse tissues [31]. Although the extent of A-to-I editing was low (less than 5% across all adenosines analyzed), targeted adenosines were at positions predicted to influence the biogenesis and function of miR-22. This raises the possibility that RNA editing may be generally important in miRNA gene function [31]. In this study we have systematically investigated the presence of RNA editing in miRNAs.

## Results

To search for RNA-editing sites in human miRNAs, PCR product sequencing was performed from matched total cDNA and genomic DNA isolated from adult human brain, heart, liver, lung, ovary, placenta, skeletal muscle, small intestine, spleen and testis. Primers were designed to amplify pri-miRNA sequences flanking all 231 human miRNAs in miRBase [6]. Of these, 99 miRNA containing sequences were successfully sequenced in both directions and from duplicate PCR products from total cDNA of at least one tissue. Total cDNA sequence traces were compared with genomic DNA sequence traces from the same individual, and A-to-I editing was identified as an A in the genomic DNA sequence compared with a novel G peak at the equivalent position in the total cDNA sequence.

**Figure 1:**
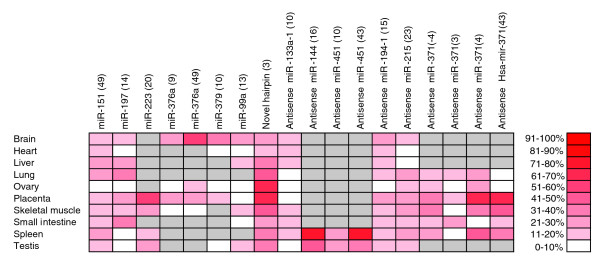
A-to-I RNA editing of miRNA precursors in human tissues. The extent of A-to-I editing at each editing site is indicated by the color scale. Each colored box represents the average extent of editing calculated from at least two PCR product sequences, at least one of which was sequenced in both directions. Gray boxes indicate miRNAs that could not be amplified. The number in brackets after the miRNA name is the position of the edited adenosine from the 5' end of the pre-miRNA or equivalent antisense pre-miRNA from the miRNA registry.

**Figure 2:**
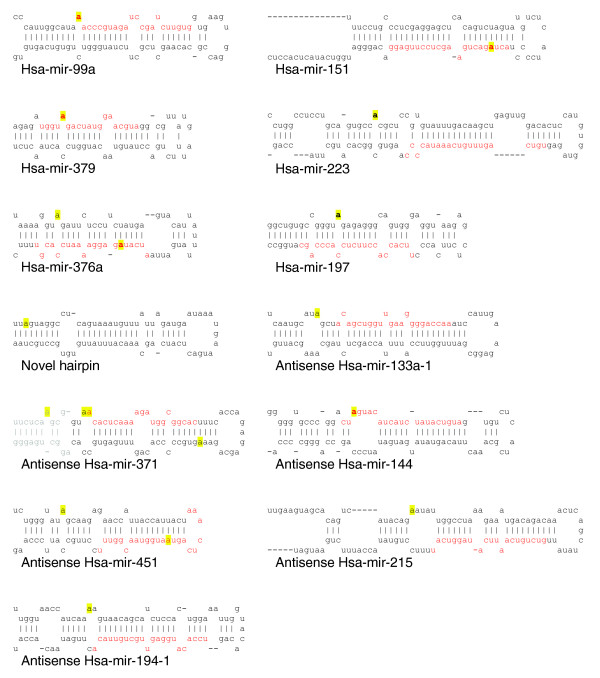
Positions of edited adenosines in human pri-miRNAs and antisense pri-miRNAs. Folded pri-miRNA structures were taken from the miRNA registry [6]. Antisense pre-miRNA structures were generated from the reverse complement pri-miRNA sequence using MFOLD [38]. Mature miRNA sequences of around 22 nucleotides and antisense mature miRNA sequences of around 22 nucleotides are indicated by red letters. Edited adenosines are highlighted in yellow. In antisense Hsa-mir-371, edited adenosines were found to reside in base-paired sequence extending beyond the annotated hairpin. Additional bases are in gray.

In total, 12 of the 99 miRNA-containing sequences (13%) were subject to A-to-I RNA editing according to A-to-G differences between matched genomic DNA and total cDNA sequence traces from at least one tissue (Figure 1). These sequences were next oriented with respect to the strand of transcription of the miRNAs. In six cases the A-to-G changes were in the same orientation as the miRNA, and overlap the stem-loop structure of the miRNA, consistent with RNA editing of the pri-miRNA precursor transcript. In an additional case, A-to-I editing was observed in a novel stem-loop structure in sequence adjacent to the unedited miRNA miR-374. This novel stem-loop structure may represent a novel miRNA (Figure 2, novel hairpin). In the remaining five cases, the A-to-G changes were from the opposite strand to the miRNA (that is, U-to-C changes in the miRNA sequence). Although U-to-C editing of miRNA sequences cannot be ruled out, no editing of this type has previously been observed and no enzymes capable of catalyzing this conversion are known. The most likely explanation is that these are A-to-I edits in a transcript derived from the DNA strand complementary to the annotated miRNA gene. Consistent with this hypothesis, all of these sequences overlap, or are adjacent to, genes transcribed from the opposite strand to the annotated miRNA gene. To distinguish these sequences from the edited pri-miRNAs, these sequences are referred to here as edited antisense pri-miRNAs (Table 1). One of the antisense pri-miRNAs contains editing sites overlapping the intended miRNA (miR-144) and miR-451, a recently identified miRNA that was not deliberately included in our list of 231 miRNAs.

Collectively the 13 sequences were edited at 18 sites. Ten out of the 13 were edited at a single site. miR-376a and antisense miR-451 were each edited at two sites, and antisense miR-371 was edited at four sites. The extent of editing varied with editing site and with tissue, ranging from around 10% (for example, miR-151 in multiple tissues) to around 70% (antisense miR-371 in placenta). Overall, the levels of RNA editing observed were considerably higher than the approximately 5% editing previously reported for the -1 position of miR-22 [31]. Editing of miR-22 was not detectable by our method, presumably because the low levels of editing of this miRNA fall below our limits of detection. All miRNAs were found to be edited in multiple tissues, with the extent of editing varying from tissue to tissue (Figure 1).

All novel A-to-I editing sites were found within the dsRNA stems of the predicted stem-loop structures (Figure 2). Of the seven editing sites in pri-miRNAs, four were in the 22-nucleotide mature miRNA. Three of these were within nucleotides 2 to 7, which are thought to be important for conferring binding-site specificity between the miRNA and its target sites [3]. Five out of seven editing sites in pri-miRNAs were at single nucleotide A:C mismatches flanked by paired bases. Similarly, five out of seven editing sites were in 5'-UAG-3' trinucleotides. These results are consistent with local structural and sequence preferences of RNA editing determined from A-to-I editing sites in inverted repeat sequences [25]. Three of the ten editing sites in antisense pri-miRNAs were in 5'-UAG-3' trinucleotides. Six of the ten editing sites were at A:C mismatches. Only one was at a single A:C mismatch, however, with the remainder at extended mismatches involving more than one consecutive nucleotide.

## Discussion

We have identified novel A-to-I editing sites in six out of 99 pri-miRNAs, indicating that at least 6% of all human miRNAs may be targets of RNA editing. We were only able to detect relatively high levels of editing, as illustrated by our failure to detect editing of miR-22, so this estimate is probably a conservative one. Moreover, our method is not strand specific, and cannot distinguish multiple overlapping transcripts from the same genomic locus. Thus, in regions of transcriptional complexity, it is likely that the sensitivity of our assay will be reduced. For example, even miRNAs that are 100% edited would appear to be unedited if transcribed at low levels compared with an unedited overlapping transcript from the opposite strand. We may also be unable to detect RNA editing if it occurs subsequent to the processing of the pri-miRNA (for example, by splicing) such that the binding sites for the PCR primers are removed.

In addition to the edited pri-miRNAs, six antisense pri-miRNA transcripts derived from the opposite strand to the annotated miRNA were subject to A-to-I editing. There are many potential explanations for apparent editing on the opposite strand to the annotated miRNA. One possibility is that these sequences are actually due to U-to-C editing of the pri-miRNA. There are, however, no known U-to-C RNA editing enzymes capable of catalyzing such a reaction, and despite extensive searches for RNA editing sites, only a single U-to-C RNA editing site has been reported [32]. It is therefore more likely that these sequences represent an edited transcript from the opposite strand to the annotated miRNA. These transcripts could be another miRNA transcribed and processed from the genomic strand opposite the annotated miRNA, or they could be some other class of transcript, for example the intron of a gene overlapping the annotated miRNA but transcribed from the opposite DNA strand. Alternatively, these may be pri-miRNAs that have been incorrectly annotated to the wrong strand of the genome.

To evaluate the possibility that the edited antisense pri-miRNAs are due to incorrect annotation of miRNAs to the wrong genomic strand, we examined previous experimental data obtained for these miRNAs. One of the edited antisense pri-miRNA sequences is derived from the DNA strand opposite the computationally predicted miR-215 [33]. The method used to predict miR-215 successfully predicted 81 out of 109 known miRNAs from a reference set, but around 20% (17/81) were predicted on the wrong strand of the genome [33]. Our data and the direction of overlapping transcripts suggest that miR-215 may have been annotated to the wrong genomic strand.

An edited antisense miRNA sequence was also derived from the DNA strand opposite experimentally verified miRNA miR-133a [34]. This miRNA is present in the genome in two copies (miR-133a-1 and miR-133a-2). Copy miR-133a-2 is hosted within a gene transcribed in the same direction as the annotated miRNA gene. In contrast, copy miR-133a-1 overlaps a gene transcribed from the opposite strand. Cloning and expression analysis of miR-133a [34] provides proof that at least one copy of miR-133a is transcribed. As a result of this finding, both copies of miR-133a have been annotated according to the sequence of the cloned copy. Given the direction of overlapping transcripts, however, it remains possible that miR-133a-1 is transcribed from the opposite strand to miR-133a-2, giving rise to a different miRNA. Indeed, our results suggest that miR-133a-1 may have been incorrectly annotated. Similarly, both copies of experimentally verified miR-194 (miR-194-1 and miR-194-2) have been annotated according to the sequence of a cloned copy [34]. Our data and the presence of overlapping transcripts on the opposite strand suggest that miR-194-1 may also have been incorrectly annotated to the wrong genomic strand. In the case of both mir-133a and mir-194, the two copies would generate miRNAs that are perfectly complementary to one another. It has previously been suggested that pairs of complementary miRNAs play a role in miRNA regulation by forming miRNA:miRNA duplexes [35]. Our results suggest that RNA editing may add a further layer of regulation by disrupting complementarity in miRNA:miRNA duplexes.

A further two edited antisense miRNA sequences (antisense mir-144 and antisense mir-451) overlap miRNAs that are annotated on the basis of their similarity to mouse miRNAs, and have not been cloned or shown to be expressed by northern blotting in human tissues. The remaining antisense miRNA sequence overlaps mir-371, which has been validated by cloning and northern blotting in human tissues and is therefore correctly annotated.

The presence of edited nucleotides in pri-miRNA transcripts indicates that RNA editing occurs early in miRNA biogenesis. Subsequent processes that recognize sequence or structural features of the miRNA precursor could therefore potentially be affected by RNA editing. These include cleavage of the pri-miRNA by Drosha, export of the pre-miRNA to nucleus by exportin-5, cleavage of the pre-miRNA by Dicer, and miRNA strand selection for inclusion in the microprocessor complex. Indeed, it has recently been demonstrated that RNA editing of pri-miRNAs can result in suppression of processing by Drosha, and subsequent degradation of the unprocessed edited pri-miRNA [36]. Although it is unclear whether a miRNA that base-pairs with its target through an I:U wobble would be functional, another possibility is that RNA editing may alter target site complementarity.

To investigate the effect of RNA editing of miRNAs on target-site complementarity, we used the miRanda software [37] to predict binding sites of edited miRNAs in 3' UTRs, and compared these with the predicted binding sites of the equivalent unedited miRNAs. For each of the four pri-miRNAs with an editing site in the mature 22mer, the set of predicted targets of edited miRNAs differs from the predicted targets of edited miRNAs (Table 1). For the three miRNAs in which the edited adenosine is at a position two to seven bases from the 5' end of the miRNA (miR-151, miR376a and miR-379) over half of the targets of the edited miRNA are unique to the edited miRNA. In the case of miR-99a the difference is small, with only 5/75 (6%) target predictions differing between edited and unedited miRNAs. In all cases, the top ten predicted targets of the edited miRNA differ from the top ten predicted targets of the unedited miRNA (data not shown).

**Figure 3:**
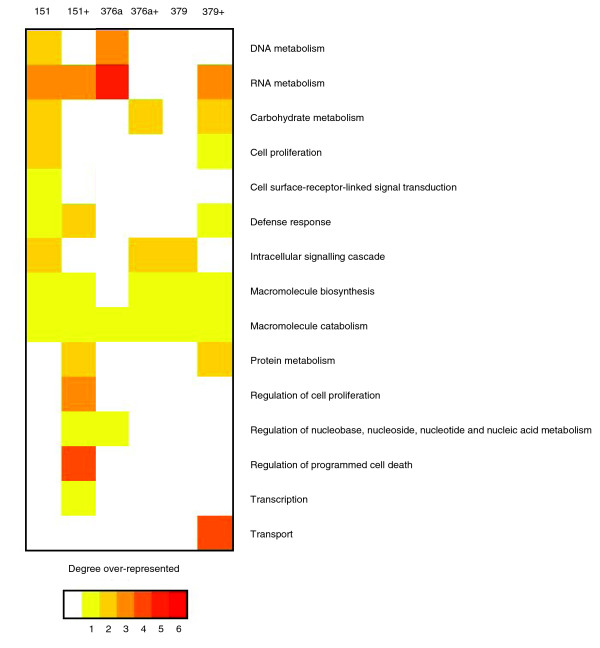
GO term comparison of edited and unedited miRNA target predictions. For each edited miRNA, GO terms from level 4 of the 'biological process' category that are over-represented in predicted targets of the unedited or edited miRNA (indicated by +) compared with all Ensembl genes were identified. All values are normalized and colored in terms of significance, with bright red cells indicating that a miRNA specifically targets genes in that GO functional class.

To gain further insight into the potential biological impact of miRNA editing, we identified Gene Ontology (GO) terms in the 'cellular process' category [38] which were over-represented in the predicted targets of edited and unedited miRNAs compared with all Ensembl genes (Figure 3). For the three miRNAs that are edited in the 5' seed region (miR-151, miR-376a and miR-379), comparison of over-represented GO terms associated with the predicted targets of edited and unedited copies reveals distinct differences (Figure 3). Of particular interest are the additional terms that become over-represented; these include regulation of programmed cell death, biosynthesis, RNA metabolism, cell proliferation and transcription (Figure 3).

RNA editing may therefore contribute to miRNA diversity by generating multiple different miRNAs from an initial pool of identical miRNA transcripts. For example, the total number of predicted targets of Hsa-mir-151 increases from 143 to 229 when taking into consideration both edited and unedited miRNAs. Editing of miRNAs may simultaneously alleviate and augment the gene-regulation effects of miRNAs by changing the concentration of individual miRNAs.

## Conclusion

We have performed the first systematic survey of RNA editing of human miRNAs. We have identified RNA editing sites in at least 6% of human miRNAs that may impact on miRNA processing, including edits that alter miRNA binding sites and contribute to miRNA diversity. Furthermore, our results suggest that some miRNA genes may have been incorrectly annotated to the wrong strand of the genome. This has implications for the interpretation of existing miRNA experiment data and future experimental design.

## Materials and methods

### Total RNA, total cDNA and genomic DNA

For the initial screen of RNA editing in ten human tissues, total RNA and matching genomic DNA from the same tissue sample was obtained for human brain, heart, liver, lung, ovary, placenta, skeletal muscle, small intestine, spleen and testis from Biochain (Hayward, USA). For each tissue, sequence data was obtained from one individual. The donor was different for each tissue type. Total cDNA synthesis was performed using random nonamers (200 ng per 20 μl reaction) with Superscript III (Invitrogen, Carlsbad, USA) according to the manufacturer's instructions.

### Sequencing of pri-miRNAs

Primers were designed to the genomic sequence in the vicinity of all 231 miRNA sequences in the miRNA registry version 7.0 [6], using primer3 [39]. PCR primer design was optimized to give PCR products of approximately 500 bp with at least 75 nucleotides either side of the predicted stem-loop structure. PCR primers were used to sequence PCR products in both directions on ABI3700 DNA sequencers. Sequence traces were quality scored using phred. Sequences with less than 70% of bases having a quality score of 20 or more were rejected. In the first stage of sequencing, duplicate PCR and sequencing was performed for each miRNA from each tissue. A miRNA was considered to be successfully sequenced if the following minimum sequence requirements were met for at least one tissue: good-quality sequence from both strands of one PCR, and good-quality sequence from at least one strand of a second PCR. Successfully sequenced miRNAs that were found to be edited were submitted to a second confirmation stage of sequencing. In the second stage of sequencing, quadruplicate PCR and sequencing was performed for each miRNA from each tissue. For each tissue, a miRNA was considered to be successfully sequenced if the following minimum sequence requirements were obtained: good-quality sequence from both strands of one PCR, and good-quality sequence from at least one strand of a second PCR. See Additional data file 1; primary sequence data is available from [40].

### Detection and quantification of RNA editing

Sequences were visualized and compared in a gap4 database. A-to-I editing was identified as a novel G peak and a drop in peak height at As in a cDNA sequence relative to the equivalent peak in the matching genomic DNA sequence. The extent of RNA editing was estimated using a modified version of the comparative sequence analysis (CSA) method [41]. Briefly, this program normalizes a cDNA sequence trace to a genomic DNA reference trace by comparison of peak heights at unedited nucleotides. The drop in peak height between the DNA reference trace and the cDNA trace at the edited nucleotide is then reported as a percentage of the peak height in the genomic DNA reference trace. For each edited miRNA, the mean extent of editing for each tissue is calculated from all cDNA sequences obtained for that tissue.

### Analysis of novel RNA editing sites

miRNA structures were obtained from the miRBase database [6]. Stem-loop structures of antisense miRNAs were generated by folding the antisense of the miRNA stem-loop sequence obtained from miRBase using MFOLD [42]. To predict edited and unedited miRNA target sites, miRanda (v3.0) [32] was used to scan the edited and unedited miRNA sequences against all human 3' UTR sequences available from Ensembl v34. The algorithm uses dynamic programming to search for maximal local complementarity alignments, which correspond to a double-stranded antiparallel duplex. The new version of the miRanda algorithm (AJ Enright, personal communication) assigns *P *values to individual miRNA-target binding sites, multiple sites in a single UTR, and sites that appear, from a robust statistical model [43], to be conserved in multiple species. The resulting targets were filtered based on *P *value (*p *< 0.001) to ensure a high degree of confidence in the predicted target sites.

### GO analysis

GO terms from level 4 of the 'cellular process' category were obtained for each human transcript from Ensembl. Over-representation for each term (*O*_term_) in a group of sequences with C terms is calculated as follows:





where *F*_1 _is the frequency of a term in the group being considered, *F*_2 _is the frequency of a term in the whole genome and *t *is the term at level *L*. GO terms with low transcript counts (< 3.0) were excluded from further analysis.

## Additional data files

The following additional data are available with this paper online. Additional data file 1 contains examples of edited sequence traces for each of the edited sites identified in this survey, and the coordinates of edited bases. Additional data file 2 contains PCR primer information, details of the initial screen of miRNAs and annotation of edited miRNAs.

## Supplementary Material

Additional data file 1 Figures containing examples of edited sequence traces for each of the edited sites identified in this survey, and the coordinates of edited bases. Click here for file

Additional data file 2 PCR primer information, details of the initial screen of miRNAs and annotation of edited miRNAs. Click here for file

## Figures and Tables

**Table 1:** Predicted targets of edited and unedited miRNAs

MicroRNA	Edited only	Unedited only	Edited and unedited
Hsa-miR-151	86	84	59
Hsa-miR-376a	74	58	71
Hsa-miR-379	79	70	75
Hsa-miR-99a	5	8	70

Target predictions were performed using the miRanda software using a probability score cut-off of *p *< 0.001. For each miRNA, the number of targets predicted for both edited and unedited miRNAs is shown against the number of targets predicted exclusively for edited miRNAs, and the number of targets predicted exclusively for unedited miRNAs.
