# Supplementary material for: RNA editing of human microRNAs
Source: Genome Biol. 2006 Apr 4;7(4):R27. doi: 10.1186/gb-2006-7-4-r27 (PMC1557993; doi:10.1186/gb-2006-7-4-r27)
Supplement: Additional data file 1 — Figures containing examples of edited sequence traces for each of the edited sites identified in this survey, and the coordinates of edited bases. [file gb-2006-7-4-r27-S1.doc]

**Supplementary Figure1.**

The following pages show examples of edited sequence traces for all novel editing sites discovered in this survey. In each case, the two upper traces are forward and reverse cDNA sequences and the two lower sequence traces are the corresponding forward and reverse genomic DNA sequences. The position of edited bases is shown in relation to the start of the annotated pre-miRNA hairpin, and corresponds to Figures 1 and 2. The genomic coordinate of each edited base is also shown.

**Hsa-mir-151**

Position of edit in miRNA: 49

Position of edit in genome: chr8:141,811,886


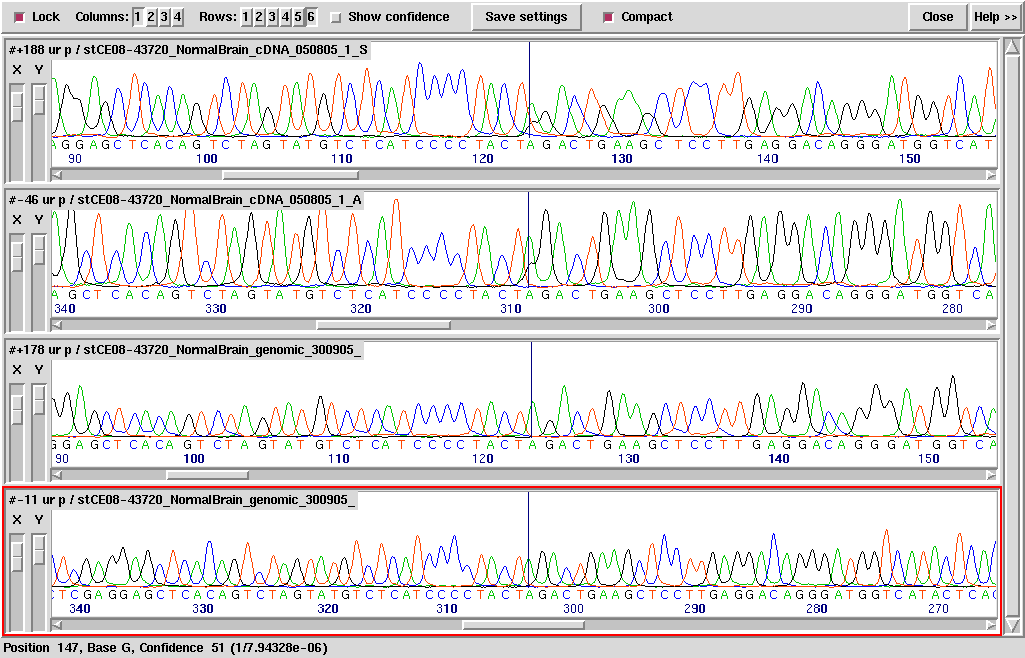


**Hsa-mir-197**

Position of edit in miRNA: 14

Position of edit in genome: chr1:109,853,570


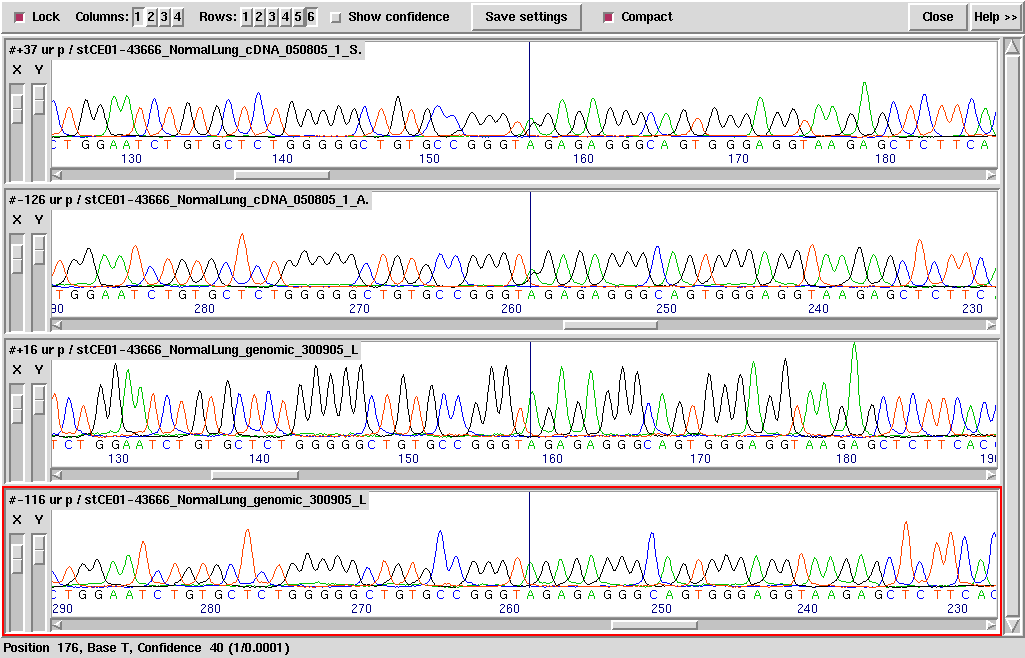


**Hsa-mir-223**

Position of edit in miRNA: 20

Position of edit in genome: chrX:65,021,752


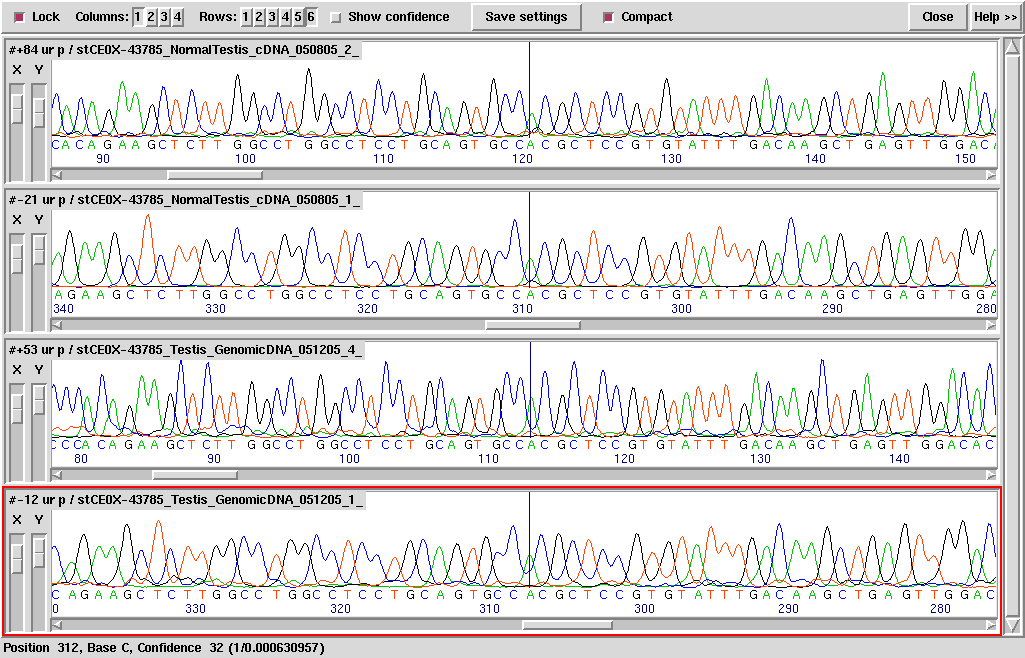


**Hsa-mir-376a**

Position of edit in miRNA: 9

Position of edit in genome: chr14:100,576,880


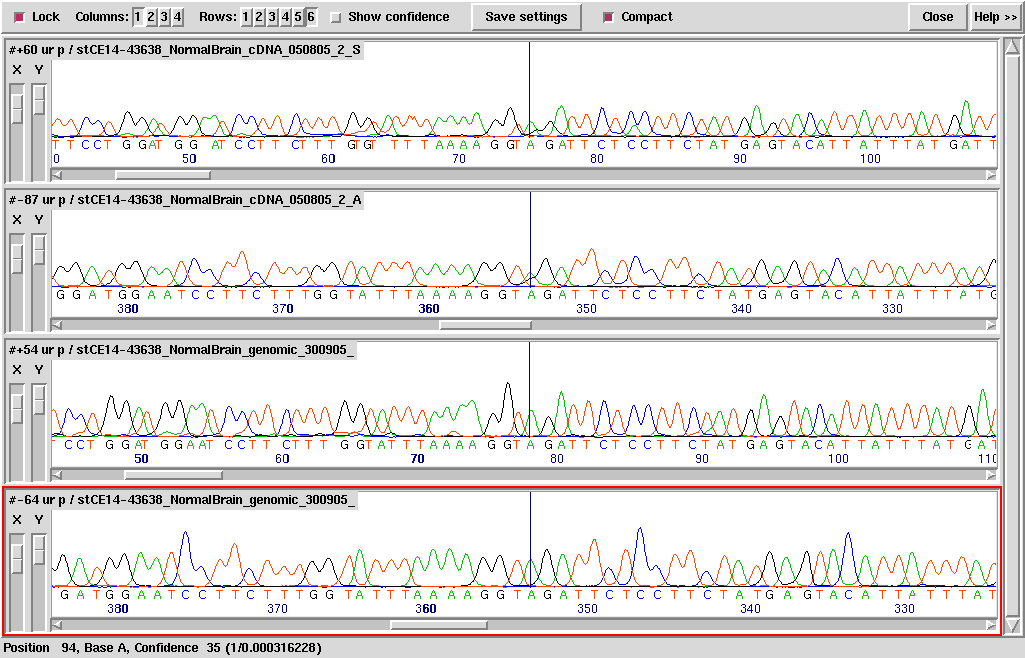


Position of edit in miRNA: 49

Position of edit in genome: chr14: 100,576,920


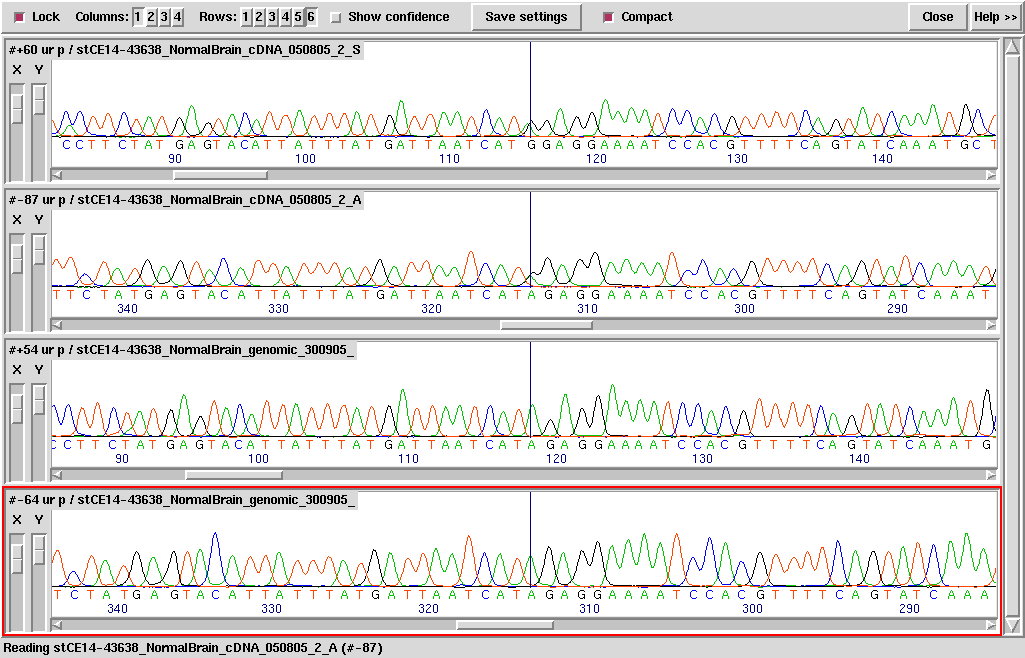


**Hsa-mir-379**

Position of edit in miRNA: 10

Position of edit in genome: chr14: 100,558,165


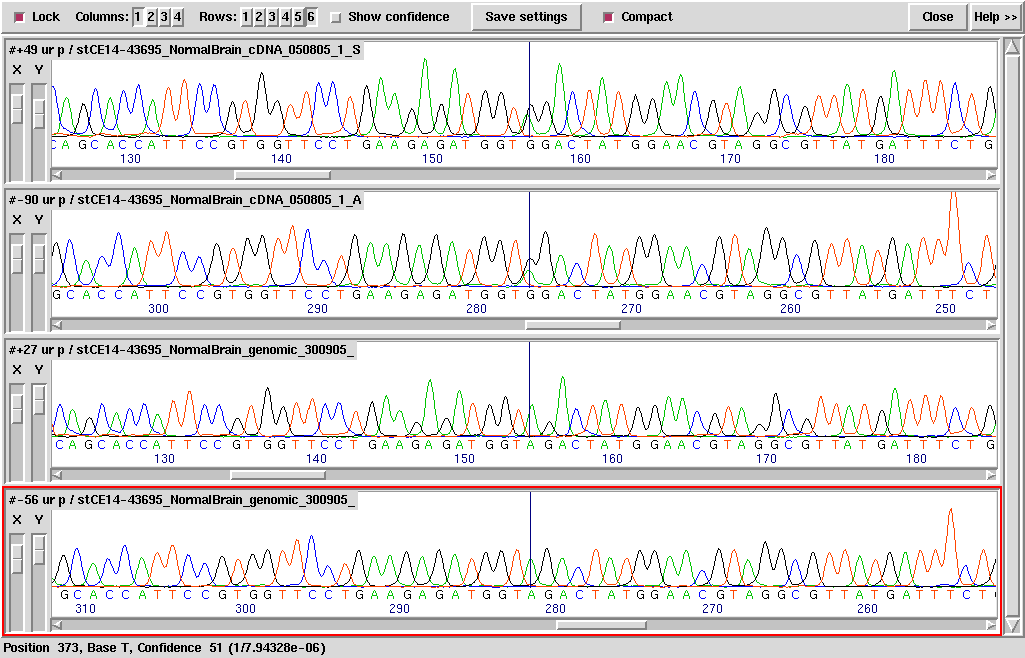


**Hsa-mir-99a**

Position of edit in miRNA: 10

Position of edit in genome: chr21:16,833,292


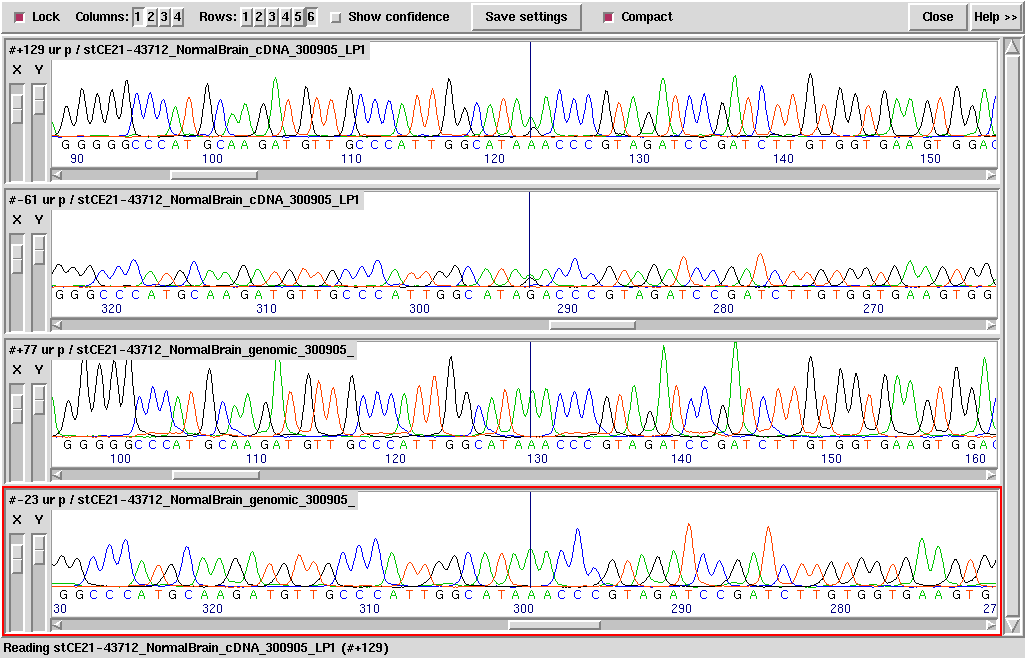


**Novel Hairpin**

Position of edit in miRNA: 3

Position of edit in genome: chrX:73,290,067


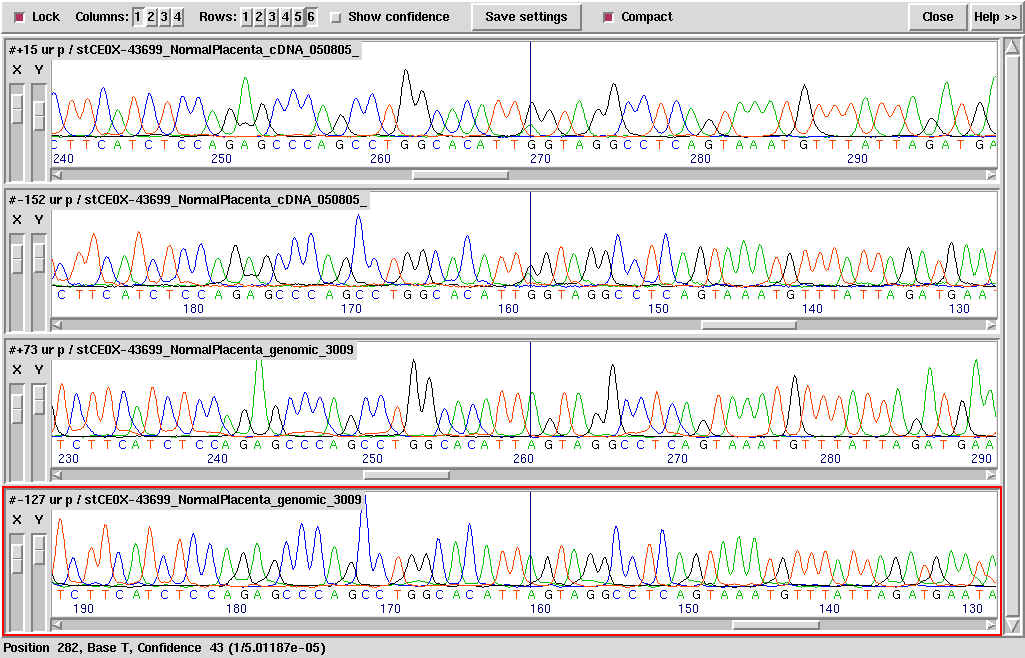


**Anti-sense Hsa-mir-133a-1**

Position of edit in miRNA: 10

Position of edit in genome: chr18:17,659,666


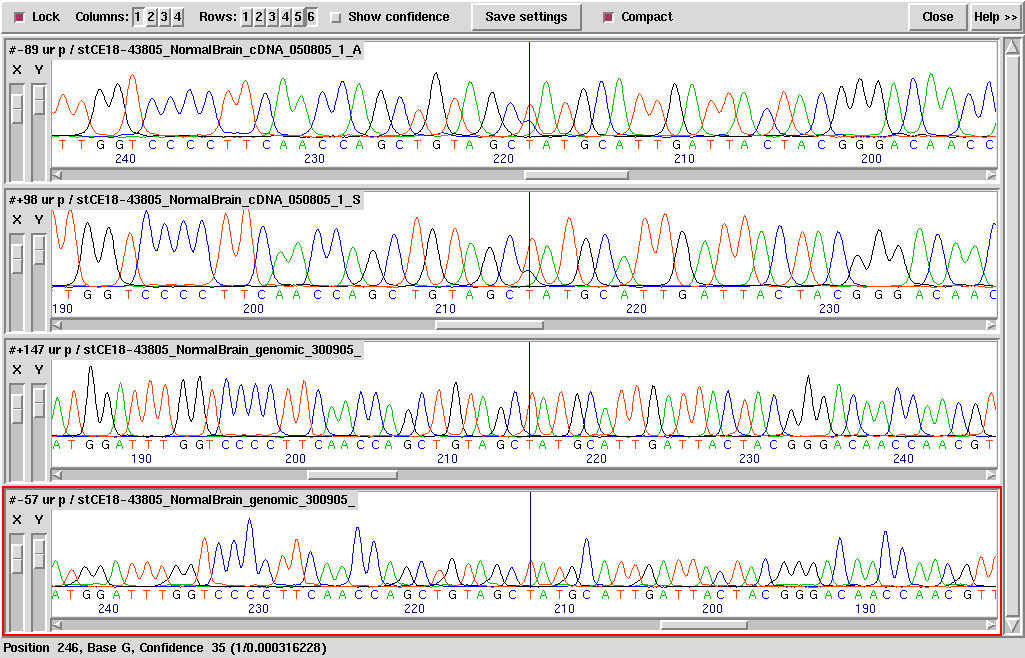
 Sequence trace is in the orientation of the annotated miRNA, edits appear as T > C changes.

**Anti-sense Hsa-mir-144**

Position of edit in miRNA: 16

Position of edit in genome: chr17: 24,212,692


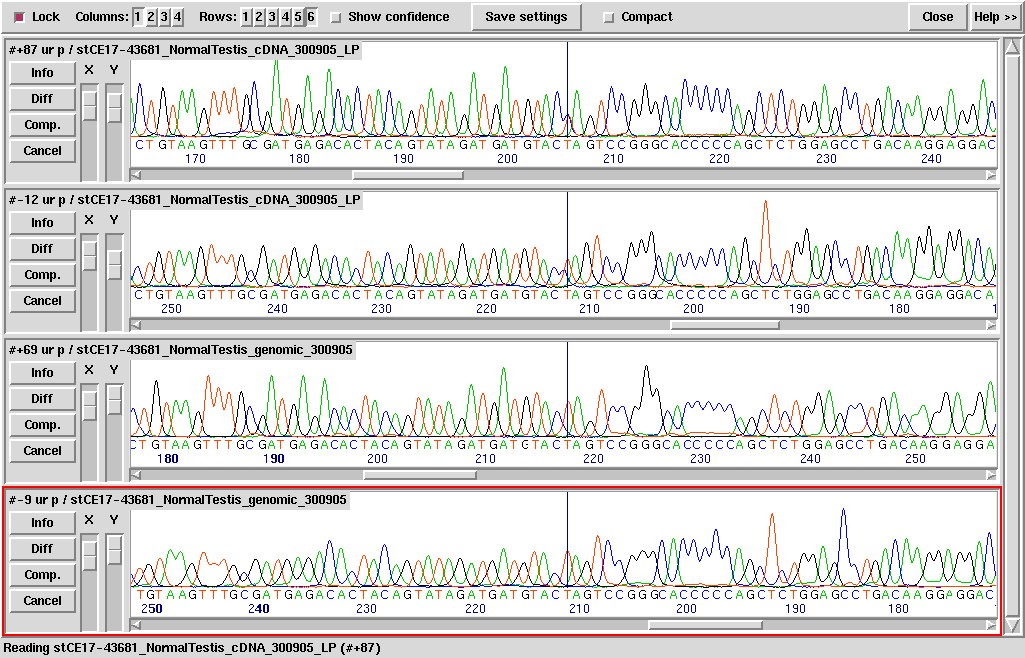
 Sequence trace is in the orientation of the annotated miRNA, edits appear as T > C changes.

**Anti-sense Hsa-mir-451**

Position of edit in miRNA: 10

Position of edit in genome: chr17: 24,212,555


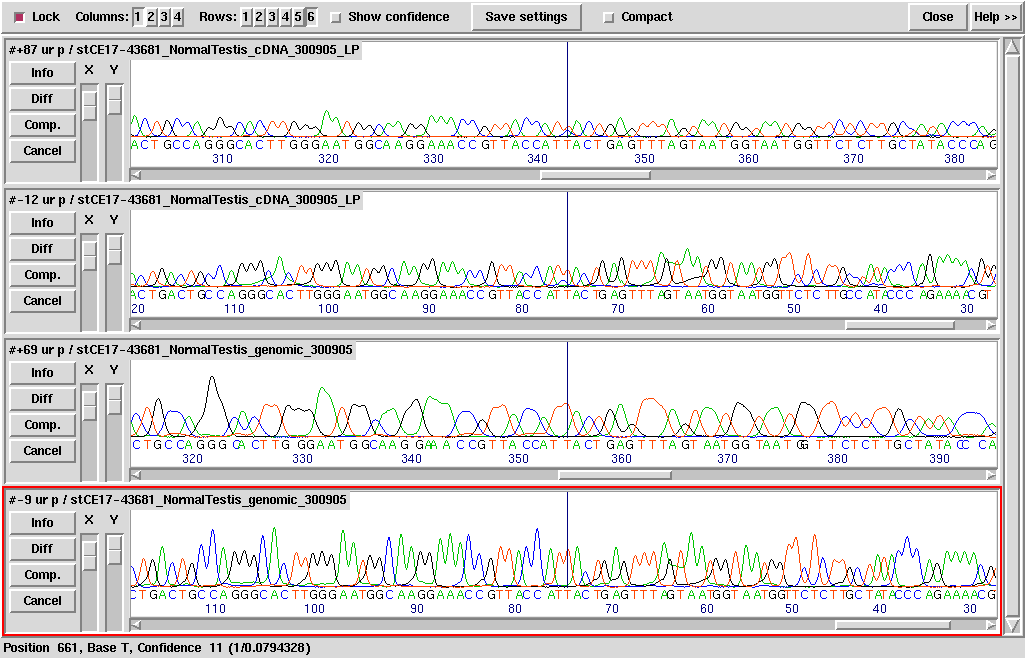


Position of edit in miRNA: 43

Position of edit in genome: chr17: 24,212,522


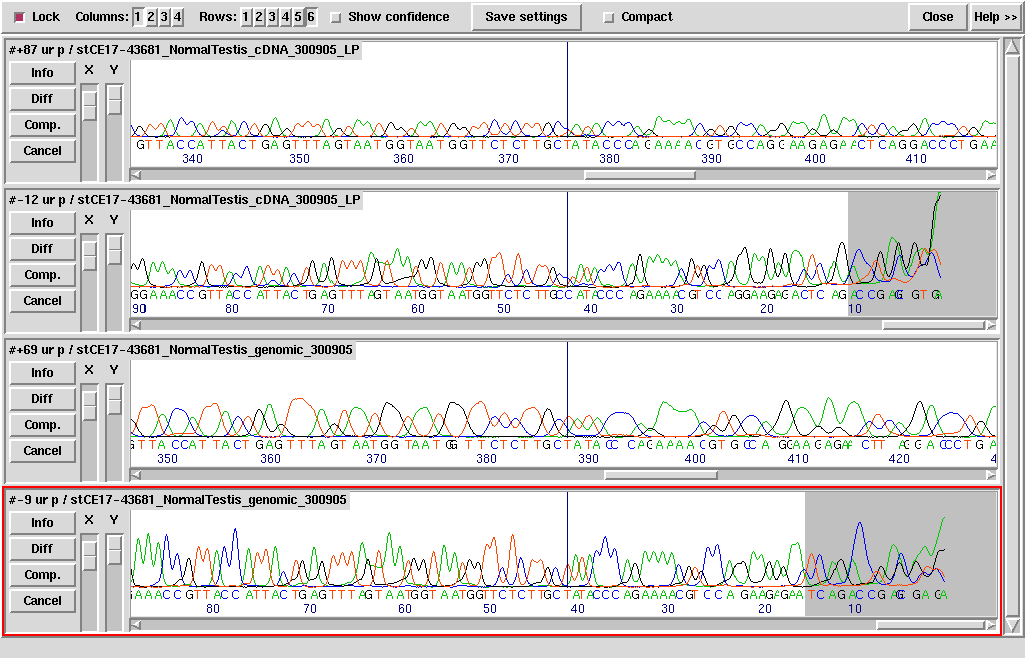
 Sequence trace is in the orientation of the annotated miRNA, edits appear as T > C changes.

**Anti-sense Hsa-mir-194-1**

Position of edit in miRNA: 15

Position of edit in genome: chr1: 216,679,908

**
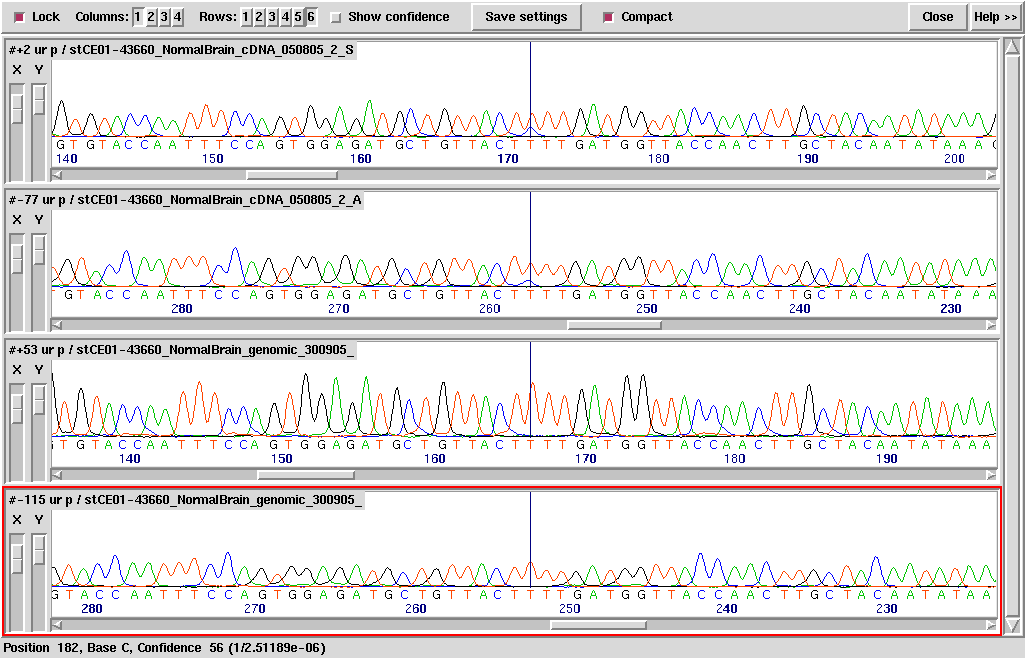
** Sequence trace is in the orientation of the annotated miRNA, edits appear as T > C changes.

**Anti-sense Hsa-mir-215**

Position of edit in miRNA: 23

Position of edit in genome: chr17: 216,679,612


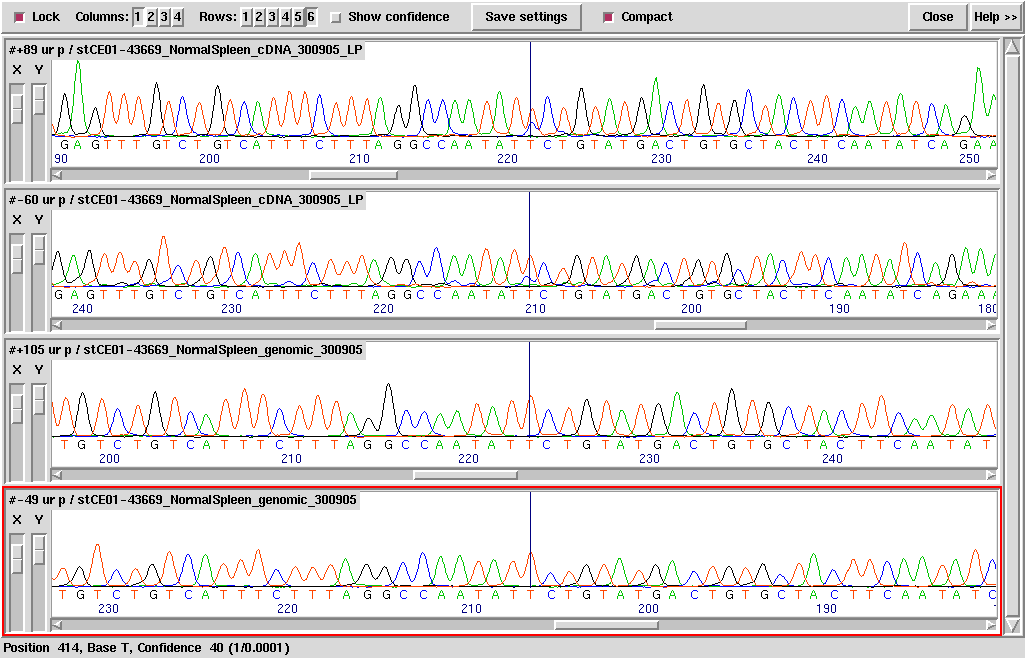
 Sequence trace is in the orientation of the annotated miRNA, edits appear as T > C changes.

**Anti-sense Hsa-mir-371**

Position of edit in miRNA: 43

Position of edit in genome: chr19: 58,982,765


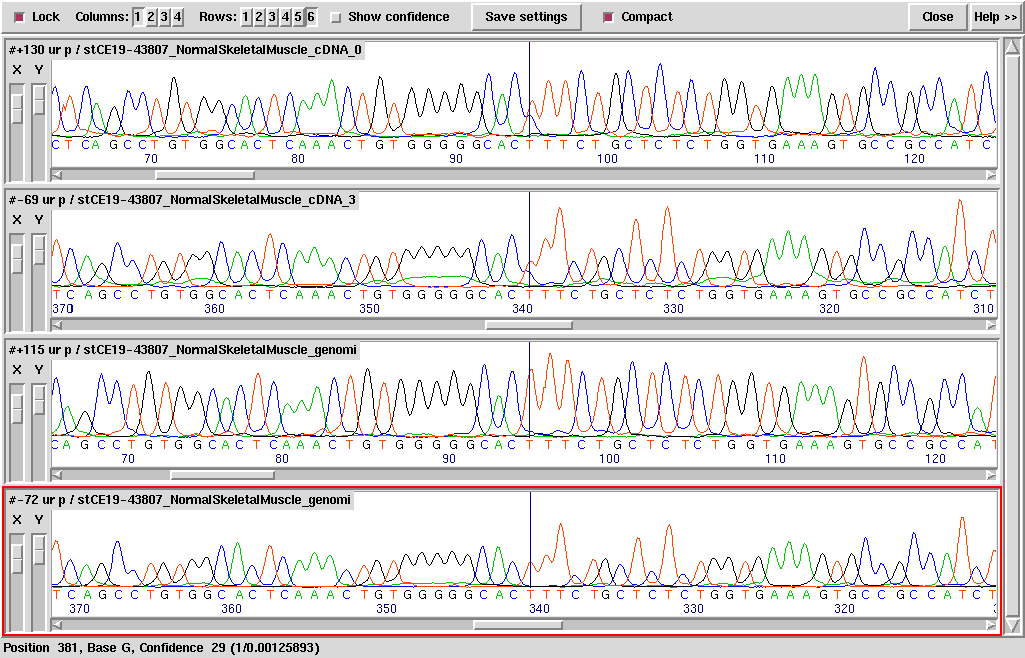


Position of edits in miRNA: -4,3,4

Position of edits in genome: chr19: 58982804, 58982805, 58982811


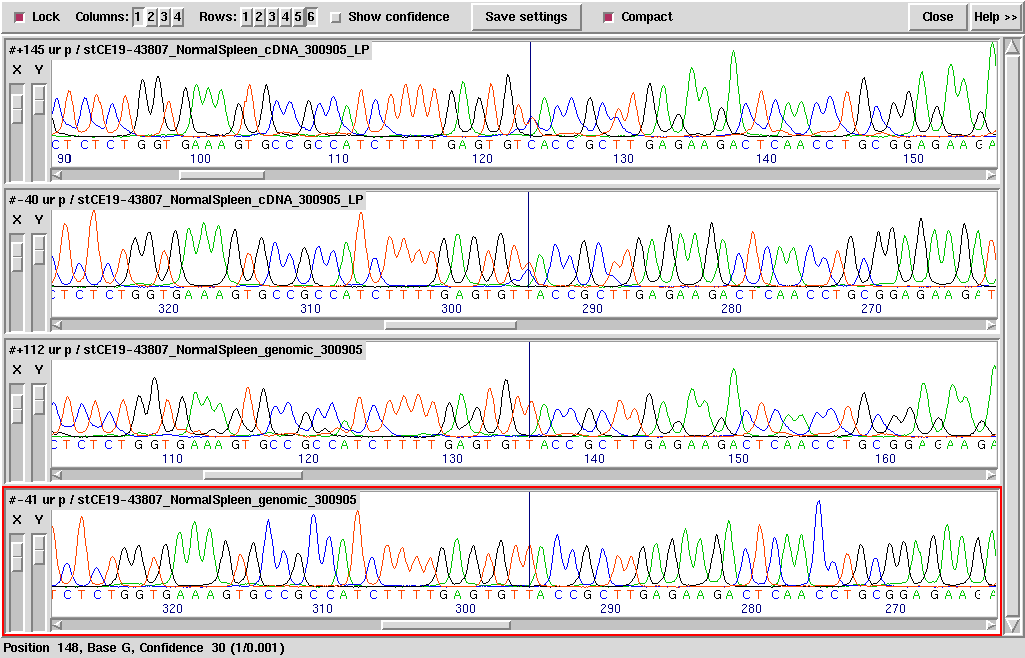
 Sequence trace is in the orientation of the annotated miRNA, edits appear as T > C changes.
